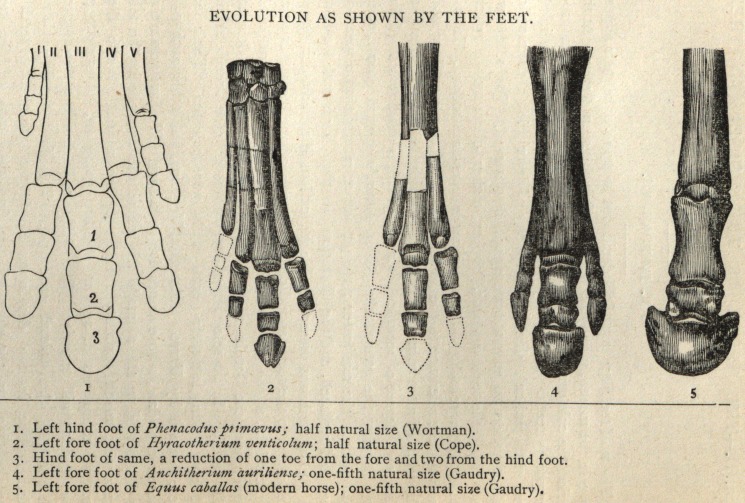# Recent Discoveries of Fossil Horses

**Published:** 1882-10

**Authors:** J. L. Wortman


THE JOURNAL

OF

COMPARATIVE MEDICINE AND SURGERY.

VOL. III. OCTOBER, 1882. No. 4.

### ORIGINAL COMMUNICATIONS.

### ART. I.—RECENT DISCOVERIES OF FOSSIL HORSES.

BY J. L. WORTMAN.

THE contributions to the knowledge of the extinct Perissodactyla, or odd-toed ungulates,* demonstrate the actual existence of types heretofore hypothetically assumed. The living representatives, the horse, tapir, and rhinoceros, constitute but a small fraction of this large order, when compared with the fossil forms already known. One of these, however, the horse, displays the most specialized structure to be found within the limits of the order.

Many years have elapsed since the first discovery in the Tertiary rocks of Europe of horse-like remains, which were regarded by paleontologists in the light of direct ancestry of the living horse. Since then the discovery of the remains of these animals in the same geological horizons in this country, by Drs. Hayden and Leidy, has strengthened the belief in the descent of the horse from very different ancestral types. Entire skeletons, obtained from the "bone beds" of the West, display their osteological characters to such an extent as to leave no doubt as to their true nature.

* The modern representatives are the horse, (1 toe), rhinoceros, (3 toes), the tapir and the little hyrax.

It is much to be regretted, however, that many of these animals have received different names from different authors, a fact very conducive to confusion. The only way to obviate this difficulty is by strict adherence to priority in the employment of a name, provided it is accompanied by a competent description, and the use of such characters as will distinguish the animal named from its nearest allies. In order to help make the subject clear, I will give the names of a few animals that have been discovered during the past forty years.

In 1841 Prof. Richard Owen decribed the remains of a Lophiodon-like* animal from the London clay of Eocene age, to which he gave the name *Hyracotherium*, †. Subsequently he described a nearly allied genus, from the same deposit, under the name *Pliolophus*, ‡. In *Hyracotherium* the molar and premolar teeth are different, both above and below. In *Pliolophus* the last, or fourth inferior premolar, is like the first true molar, a character which separates the two genera satisfactorily. The specimens described by Prof. Owen do not display clearly the number of digits either possessed, but he expresses the opinion that *Pliolophus* has three toes on the posterior limbs.

In 1872 Professor O. C. Marsh discovered in the deposits of the Eocene age, three forms of fossil horse, these were distinguished as *Orohippus*, *Eohippus* and *Orotherium*. The last two forms are probably identical with Owen's *Pliolophus*, as *Pliolophus* is nearly allied to *Hyracotherium*, we may say that Hyracotherium was the most generalized form of the fossil horse known till recently. An other and near ally to the above form has been discovered by Prof. Cope

* The Lophiodons were first described by Cuvier. They were allied to the tapir. They derive their name from the structure of the true molars, which have their crowns crossed transversely by two crests or ridges of dentine, covered with a layer of enamel. The last lower molar has also a small posterior lobe. The premolars are more simple in structure and compressed, resembling the first premolars of the tapir. The upper molars also resemble those of the tapir, but approach, in some respects, those of the rhinoceros. The diastema, or toothless interval between the canine and pre-molar teeth, was much shorter than in the tapir. Several species have been described from the Eocene of France and England, but little is known of the skull or skeleton. No true Lophiodon is yet certainly known in this country.—O. C. MARSH.

† Transactions London Geological Society, 1841, pp. 203–208.

‡ Loc. Cit., pp. 54–72, 1858.

in New Mexico. This genus Cope has named *Systemodon,** and assigns as his reasons for separating it from *Hyracotherium* the circumstance that it displays no diastemata spaces behind the superior canines, while in the latter there are two. This fossil (from New Mexico) was first described by him under the name *Hyrocotherium tapirinum*,† but the discovery of better specimens demonstrates its claim to the rank of a new genus.

We now turn to the description of a still older form than the above made known by Prof. Cope in 1873.‡ This is the *Phenacodus.* Its systematic position in the mammalian class was involved in considerable uncertainty till the discovery of the greater part of the skeletons of two distinct species of this genus by the writer in the Wyoming Wasatch during the summer of 1881, which afforded Prof. Cope the means of determining its true position and elucidating the many important and interesting points its osteology teaches. Prior to the discovery of these skeletons no characters had been found among the Perissodactyla that would justify the systematist in dividing the group into more than a *single* sub-order; but it is now necessary to separate it into *two* sub-orders. These Prof. Cope designates the *Condylarthria* and the *Diplarthria*.§ The characters on which this division reposes are to be found in the astragalus || and its manner of articulation.

It is sufficient to say now that the two sub-orders are well distinguished and that their discovery furnishes another link and one long sought in the evolution of the horse from earlier forms.

The *Phenacodus* of Cope, is the type genus of the *Condylarthria.* It possesses five well developed toes in functional use on all the feet, of which the first is the smallest; the medium is the largest, and is symmetrical within itself. The feet are considerably shortened, and were probably semiplantigrade; in fact, the feet of this animal constitute an approach to the Amblypoda. The dental formula is: Incisors, 3-3, 3-3; canines, 1-1, 1-1; premolars, 4-4, 4-4; molars,

---

* Rept. U. S. Geol. Surv., W. 100th Mer., Capt. G. M. Wheeler, Pt. ii., Vol. iv., p. 263.

† Paleontological Bulletin, No. 34, Dec., 1881, p. 177.

‡ Paleontological Bulletin, No 17, October, 1873, p. 3.

§ Paleontological Bulletin, No. 34, December. 1881, p. 178.

|| A bone of the hock corresponding to the ankle-bone of man.

3-3, 3-3, 4-4; that is, 44 functionally developed teeth. The molars are of the simple four-lobed pattern, resembling in this respect the suilline Artiodactyla, or hogs and peccaries; in fact, on this account it is a matter of some surprise that the animal should turn out to belong to the Perissodactyla. Professor Cope has described five genera of this type.*

---

* As the *Phenacodontidæ* (Cope) plainly present us with this hypothetical condition, both as regards the teeth and the number of digits on each limb, they cannot be regarded otherwise than as the primitive ancestors of the succeeding members of this important and once populous order. There has probably been no discovery among the ungulates since the finding of the *Amblypoda* that has proved equal in interest and importance to the discovery of this group. The descent of all the ungulates from the *Amblypoda* has been held by Prof. Cope for some time, but that it took place from any *known* genera of this order, the comparatively specialized condition of the teeth of the latter distinctly forbids This moderate complexity of the teeth among Eocene mammals is a striking exception, especially when associated with such a low grade of organization of other parts as we find in these animals. The explanation of this fact must, in my judgment, be sought for in their large size and in the possession of powerful canine teeth, which insure them greater immunity from the attacks of fierce carnivorous contemporaries. With these means of defense, they could take up their abode where food better adapted to their wants was furnished. Hence we can with perfect consistency look for a rapid modification of these organs, accompanied with slight change in others. In order to make the connection complete between them and the Phenecodonts, there should yet be found an Amblypod with bunodont molars, reduced canines and a more elongated foot. An approach to this condition, as far, at least, as the molars are concerned, is found in a new form recently described by Prof. Cope, under the name Manteodon (prophecy tooth). The *Amblypoda*, says Professor Cope in his Report on Capt. Wheeler's Survey (W. 100th Mer.), are as yet confined to the Eocene period exclusively, and are found both in Europe and this country. In points of affinity to the hoofed orders generally they occupy an interesting and important position; being in all probability the oldest, and affording the most generalized condition known among the ungulates. The brain capacity is exceedingly small in proportion to the size of the other parts of the skeleton, and from casts made from the brain case itself we are warranted in assigning these animals a position among the lowest mammalia; they are lower in brain development even than some of the *Marsupials*. The feet are very short, are provided with five fully developed toes, and have their entire plantar and palmar surfaces

The Meniscotheriidæ has been recently established for the reception of the single genus Meniscotherium, discovered by Prof. Cope in the Wasatch beds of New Mexico, and described by him in his report to Captain Wheeler. The recent discovery of the bones of the feet shows that they display the characteristic peculiarities of the *Condylarthria,* to which group it must be referred. Its digital formula is unknown, hence we must rely upon the specialized crescentoid pattern of the molars for the family definition. It is proper to remark here that reduction in digits in the Perissodactyla is usually accompanied by specialization of the molar teeth. In this case, therefore, I would venture the prediction that its digital formula will be

---

applied to the ground, as in the modern bears. The astragalus is greatly

Right hind foot of a species of Coryphodon (Amblypod), one-half natural size (Cope).

flattened from above downward, and is primitive and characteristic. It displays on its inferior surface flattened articular facets for both navicular and cuboid bones, which share the articulation about equally. On the superior part, the surface articulating with tibia, it is almost flat, a condition which must have rendered the ankle joint capable of very little movement, and giving to these animals a peculiarly awkward and shambling gait It is not difficult to perceive that these small-brained, five-toed, and plantigrade *Amblypoda* could easily have furnished a starting point for both the *Artiodactyla* and *Perissodactyla,* and, as we have good reasons to believe, did give origin to the *Proboscidea* or elephants.

found to be 4-3, with the outer toes somewhat reduced. The value of the digital formula, as a character, in the definition of the families of the Perissodactyla is of high standard. This may, likewise, be said of the relations of the molar and premolar teeth, but in a less degree. The tubercular, or crescentoid structure of the molars, however, is capable of such intergradation, which increase of our knowledge demonstrates, that it must be accepted as provisional only, and not entitled to rank equal in value to either of the other two characters in defining the family.

The genealogy of the horse as now indicated is as follows:

*Diplarthria* . . . {

Equus, — Equus,

Protohippus, — Hippotherium,

Anchippus, — Paloplotherium,

Anchitherium,

Mesohippus,

Lambdotherium,

Oligotomus,

Hyracotherium,

Systemodon,

*Condylarthria* { Meniscotherium,

Phenacodus,

AMBLYPODA, — Hyodonta.

The cause of this digital reduction is an interesting inquiry Bunodonts* as a rule are dwellers in swamps and forests and live on nuts, berries, and roots. If they are compelled to forsake their natural habitat and live in the open field, either modifications or extinction will follow. Once in the open field speed becomes a desideratum as a condition of safety, and the foot with a reduced number of digits possesses many advantages over the polydactyle one.

Prof. Cope has shown (*American Naturalist*, April, 1881), that in plantigrade quadrupeds the extremities of the toes are arranged in a semi-circle, when they are all applied to the ground. In the act of running the heel and wrist are raised, throwing the weight of the body upon the median digits. An infinite repetition of this posture in digitigrade animals unable to withstand the attacks of their ene-

* Teeth of complicated structure, with high and uniformly broadened rowns, the face presenting a complex folding of the enamel plates.

EVOLUTION AS SHOWN BY THE FEET.

1. Left hind foot of *Phenacodus primævus*; half natural size (Wortman).
2. Left fore foot of *Hyracotherium venticolum*; half natural size (Cope).
3. Hind foot of same, a reduction of one toe from the fore and two from the hind foot.
4. Left fore foot of *Anchitherium aurilense*; one-fifth natural size (Gaudry).
5. Left fore foot of *Equus caballas* (modern horse); one-fifth natural size (Gaudry).

mies and whose only escape was in flight, the strengthening of the median digits, and the consequent reduction of the outer ones, would follow according to the law of use and disuse of parts. This subtraction of toes has progressed step by step until the modern one-toed horse has been reached.

## Figures and Tables

**Figure f1:**
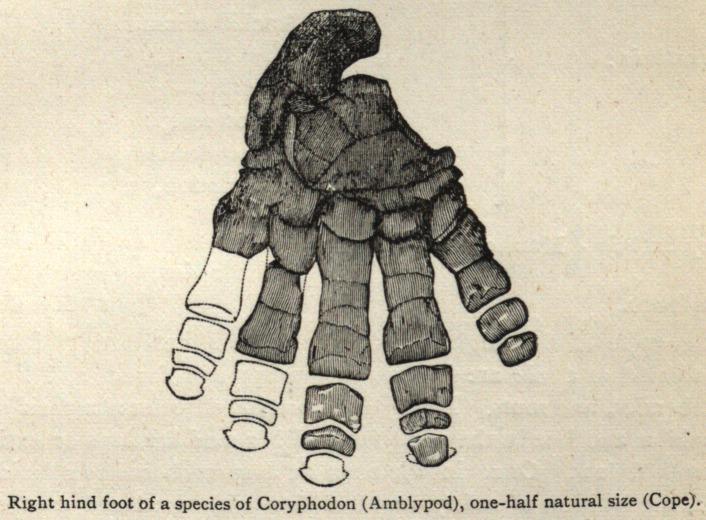


**Figure f2:**
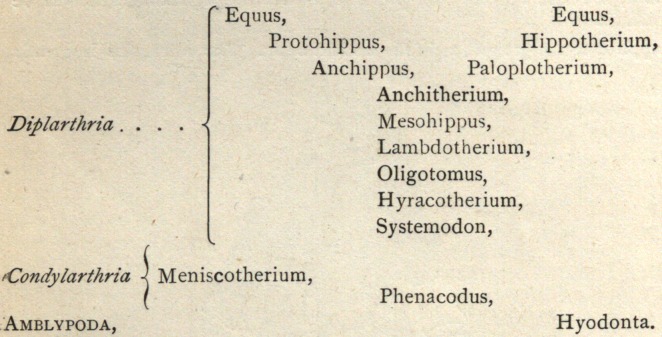


**Figure f3:**